# *Drosophila* linker histone H1 coordinates STAT-dependent organization of heterochromatin and suppresses tumorigenesis caused by hyperactive JAK-STAT signaling

**DOI:** 10.1186/1756-8935-7-16

**Published:** 2014-07-28

**Authors:** Na Xu, Alexander V Emelyanov, Dmitry V Fyodorov, Arthur I Skoultchi

**Affiliations:** 1 Department of Cell Biology, Albert Einstein College of Medicine, 1300 Morris Park Avenue, Bronx, NY 10461, USA

**Keywords:** Heterochromatin, Linker histone H1, JAK-STAT signaling, Tumor suppressor, Melanotic tumors, *Drosophila melanogaster*

## Abstract

**Background:**

Within the nucleus of eukaryotic cells, chromatin is organized into compact, silent regions called heterochromatin and more loosely packaged regions of euchromatin where transcription is more active. Although the existence of heterochromatin has been known for many years, the cellular factors responsible for its formation have only recently been identified. Two key factors involved in heterochromatin formation in *Drosophila* are the H3 lysine 9 methyltransferase Su(var)3-9 and heterochromatin protein 1 (HP1). The linker histone H1 also plays a major role in heterochromatin formation in *Drosophila* by interacting with Su(var)3-9 and helping to recruit it to heterochromatin. *Drosophila* STAT (Signal transducer and activator of transcription) (STAT92E) has also been shown to be involved in the maintenance of heterochromatin, but its relationship to the H1-Su(var)3-9 heterochromatin pathway is unknown. STAT92E is also involved in tumor formation in flies. Hyperactive Janus kinase (JAK)-STAT signaling due to a mutation in *Drosophila* JAK (Hopscotch) causes hematopoietic tumors

**Results:**

We show here that STAT92E is a second partner of H1 in the regulation of heterochromatin structure. H1 physically interacts with STAT92E and regulates its ectopic localization in the chromatin. Mis-localization of STAT92E due to its hyperphosphorylation or H1 depletion disrupts heterochromatin integrity. The contribution of the H1-STAT pathway to heterochromatin formation is mechanistically distinct from that of H1 and Su(var)3-9. The recruitment of STAT92E to chromatin by H1 also plays an important regulatory role in JAK-STAT induced tumors in flies. Depleting the linker histone H1 in flies carrying the oncogenic *hopscotch*^*Tum-l*^ allele enhances tumorigenesis, and H1 overexpression suppresses tumorigenesis.

**Conclusions:**

Our results suggest the existence of two independent pathways for heterochromatin formation in *Drosophila*, one involving Su(var)3-9 and HP1 and the other involving STAT92E and HP1. The H1 linker histone directs both pathways through physical interactions with Su(var)3-9 and STAT92E, as well with HP1. The physical interaction of H1 and STAT92E confers a regulatory role on H1 in JAK-STAT signaling. H1 serves as a molecular reservoir for STAT92E in chromatin, enabling H1 to act as a tumor suppressor and oppose an oncogenic mutation in the JAK-STAT signaling pathway.

## Background

The genomes of eukaryotes are packaged into a nucleoprotein complex called chromatin [1,2]. Compaction of the DNA is achieved primarily through its association with a small family of proteins called histones. There are five major classes of histones: the core histones H2A, H2B, H3, and H4 and the linker histone usually referred to as H1. The nucleosome core particle is the basic repeating unit of chromatin in which approximately 145 bp of DNA is wrapped around an octamer of the four core histones. The linker histone H1 binds to the nucleosome core particle near the site at which DNA enters and exits the core particle, organizing an additional of approximately 20 bp of DNA to form the chromatosome [3]. The binding of H1 stabilizes the core particle and facilitates folding of nucleosome arrays into higher order structures [4,5].

The structure of chromatin is dynamic and undergoes changes in compaction during the cell cycle and during development. Importantly, among the five classes of histones, the H1 linker histone exhibits the greatest mobility, shuttling between the chromatin and the nucleoplasm with a residence time in chromatin of approximately 3 min [6,7]. Within the nucleus, chromatin exhibits variable levels of packaging. Chromatin is organized into densely packaged, generally silent regions called heterochromatin and more loosely packaged, transcriptionally active euchromatin [8]. Heterochromatin identity is established through modifications of epigenetic landscape of the genome and recruitment of specialized protein factors [9,10]. An important breakthrough in our understanding of the molecular basis for heterochromatin formation came with the discovery that the H3 histones in heterochromatin are modified by methylation on lysine 9 in the H3 N-terminal tail. This modification is catalyzed by the histone methyltransferase Su(var)3-9 [11] and it is recognized and bound by heterochromatin protein 1 (HP1) [12]. Recently, we reported that the linker histone H1 in *Drosophila* is also required for heterochromatin formation [13] and that it recruits Su(var)3-9 to heterochromatin by directly interacting with it [14]. H1 also has been shown to interact with HP1 [14-17].

Another factor that has been linked to heterochromatin stability in *Drosophila* is the DNA binding protein STAT92E [18-20]. Flies have a single STAT (STAT92E) and a single Janus kinase (JAK) that together constitute the JAK-STAT signaling pathway in *Drosophila*[21-23]. Perturbations of this pathway, including depletion of STA92E or expression of a mutant hyperactive JAK (*hop*^*Tum-l*^), lead to heterochromatin instability. The *hop*^*Tum-l*^ mutation also leads to the formation of blood cell tumors [24,25]. We show here that the H1 linker histone directly interacts with STAT92E and regulates its roles in both heterochromatin formation and tumorigenesis. Our results identify a second pathway of heterochromatin formation that is distinct from that of H1 and Su(var)3-9. Our observations also establish linker histone H1 as a tumor suppressor in flies.

## Results and discussion

### Hyperactive JAK/STAT signaling disrupts pericentric heterochromatin

Previous reports implicated the JAK-STAT signaling pathway in heterochromatin stability and heterochromatin protein1 (HP1) localization in *Drosophila. Tumorous-lethal* (*Tum-l*), an oncogenic allele of *hopscotch* (*hop*) encoding a constitutively hyperactive mutant of *Drosophila* JAK, was observed to disrupt heterochromatic silencing and HP1 localization in heterochromatin [18]. Loss of *Drosophila* STAT (STAT92E) was found to have very similar effects [19]. Based on the observation that HP1 and STAT92E interact, it was proposed that the two proteins colocalize within heterochromatin and that unphosphorylated STAT92E regulates HP1 localization and heterochromatin stability [19].

**Figure 1:**
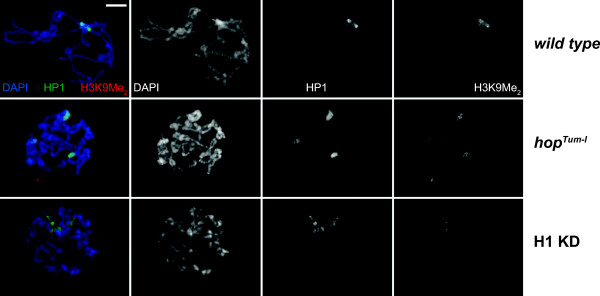
**Hyperactive JAK affects chromocenter formation in *Drosophila *polytene chromosomes.** Polytene chromosomes of salivary gland cells from L3 larvae were analyzed by indirect immunofluorescence (IF) staining with antibodies against HP1 (green) and H3K9Me_2_ (*red*). DNA was stained with DAPI (*blue*). *Top*, *wild type* polytene chromosomes have a uniform regular structure of bands and interbands with a single chromocenter characterized by overlapping intense HP1 and H3K9Me_2_ staining. *Middle*, *hop*^*Tum-l*^ polytene chromosomes have an abnormal morphology with disrupted polytene chromosome structure and dispersed HP1 foci. A single chromocenter cannot be discerned by DAPI or HP1 staining. H3K9Me_2_ staining overlaps with HP1-positive foci. *Bottom*, H1-depleted polytene chromosomes have an abnormal morphology with disrupted polytene chromosome structure and dispersed HP1 foci. A single chromocenter cannot be discerned by DAPI or HP1 staining. H3K9Me_2_ staining is strongly reduced. Scale bar represents 10 μm.

The effects of JAK-STAT signaling on heterochromatin were postulated based on low-resolution whole-mount staining of salivary glands for HP1. To examine in more detail the effects of perturbing JAK-STAT signaling on chromatin structure, we analyzed polytene chromosomes from salivary glands of *hop*^*Tum-l*^ mutant larvae. Figure 1 shows a comparison of polytene chromosomes prepared from control and *hop*^*Tum-l*^ mutant salivary glands. The overall structure of polytene chromosomes in the mutant is not severely perturbed, and they exhibit a close-to-normal pattern of bands and interbands. However, DAPI staining in *hop*^*Tum-l*^ salivary glands revealed that the polytene chromosomes lack a discernable chromocenter, the single coalesced region of heterochromatin formed from the pericentric regions of all chromosomes. This region is embedded in heterochromatin and normally exhibits intense staining for HP1. In contrast, HP1 staining in *hop*^*Tum-l*^ mutants was dispersed into several discrete foci. Interestingly, the abnormal chromocenter structure in *hop*^*Tum-l*^ animals resembles that in salivary glands of animals depleted of the linker histone H1 [13]. To directly compare effects of *hop*^*Tum-l*^ mutation and H1 depletion on salivary gland structure, we recombined two transgenes encoding an H1 RNA hairpin driven by the GAL4-responsive UAS promoter and pActin-GAL4. At 29°C, these transgenes together cause a moderate depletion of H1 in larvae (see Additional file 1: Figure S1), to approximately 30% of the wild type level. We observed that *hop*^*Tum-l*^ mutation or moderate H1 depletion in L3 larvae results in comparable defects of polytene chromosome structure (Figure 1). Therefore, we hypothesized that H1 and STAT92E may cooperate to maintain a normal polytene chromosome architecture and heterochromatin structure and function.

Interestingly, although hyperactive JAK clearly disrupts HP1 localization and formation of a single chromocenter in polytene chromosomes, it does not lead to a reduced amount of the H3K9 dimethyl mark in pericentric heterochromatin. In contrast, depletion of H1 causes a marked reduction in pericentric H3K9Me_2_ signal (Figure 1). Thus, H1 and STAT92E may share some but not all roles in regulation of heterochromatin structure and activity.

### H1 regulates localization of STAT92E in chromatin

**Figure 2:**
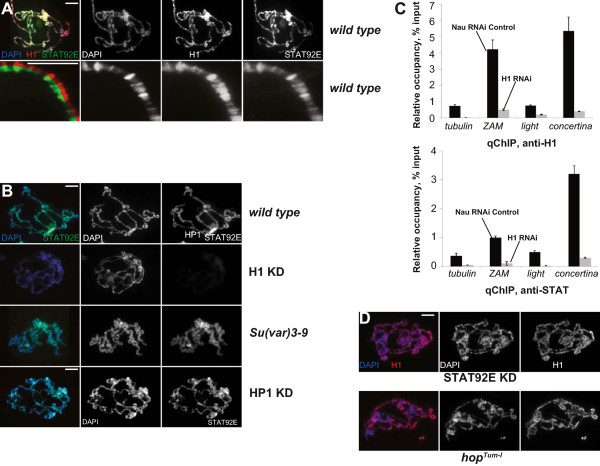
**Distribution of STAT92E in polytene chromosomes depends on H1.** Polytene chromosomes of salivary gland cells from L3 larvae were analyzed by indirect immunofluorescence (IF) staining with antibodies against H1 (*red*) and STAT92E (*green*). DNA was stained with DAPI (*blue*). *Scale bars* represent 10 μm. **(A)***Top*, genome-wide localization of H1 and STAT92E in polytene chromosomes. Localization patterns of H1 and STAT92E extensively overlap in the euchromatic arms and the chromocenter of polytene chromosomes. DNA was stained with DAPI (*blue*). *Bottom*, higher magnification view of co-localization of H1 and STAT92E in polytene chromosome arms. Merged split image illustrates that STAT and H1 exhibit nearly identical localization patterns, which correlate with polytene bands. **(B)** Genome-wide localization of STAT92E in *wild type*, H1-depleted, *Su(var)3-9*[1]*/Su(var)3-9*[2] and HP1-depleted polytene chromosomes. In H1-depleted salivary glands, the polytene chromosome structure is disrupted, and STAT92E staining is strongly reduced to barely above background. Neither *Su(var)3-9* mutation nor HP1 depletion substantially affects STAT92E localization. **(C)** The occupancy of H1 and STAT92E at regulatory regions of euchromatic (*tubulin*) and heterochromatic (*light*, *concertina*) genes and transposable element *ZAM*. The occupancy was measured by qChIP in control and H1 RNAi alleles. The ordinate indicates the amounts of specific polymerase chain reaction (PCR) products in ChIP DNA samples relative to input DNA. All qChIP experiments were performed in triplicate. Error bars, standard deviation. **(D)** Genome-wide localization of H1 in STAT92E-depleted and *hop*^*Tum-l*^ mutant polytene chromosomes. The localization pattern of H1 is not affected and is similar to that in *wild type* chromosomes (compare to A).

To begin to investigate the relationship between H1 and STAT92E, we sought to determine whether the two proteins co-localize in polytene chromosomes. By using indirect immunofluorescence (IF) staining, we found that STAT92E co-localizes with H1 in the chromocenter and throughout euchromatic arms (Figure 2A). High magnification images of polytene chromosome arms from wild type salivary glands display a strikingly similar pattern of staining for H1 and STAT92E, with both proteins most highly concentrated in bands. Split images for H1 and STAT92E show virtually complete overlap in their distributions. These observations indicate that H1 and STAT92E co-localize throughout polytene chromosomes.

To further investigate the basis for H1 and STAT92E co-localization in chromatin, we analyzed their distribution in salivary gland nuclei in which H1 was depleted by RNAi. As expected, we observed reduced H1 abundance in polytene chromosomes of H1-depleted larvae (Additional file 1: Figure S2A). Strikingly, we also found that specific STAT92E staining of polytene chromosomes, including the chromocenter and euchromatic arms, is almost completely lost upon H1 depletion (Figure 2B). In control experiments, STAT92E or H1 localization is not substantially affected in animals with a homozygous null mutation of *Su(var)3-9* or with HP1 depleted by RNAi (Figure 2B and Additional file 1: Figure S2A). The observed mis-localization of STAT92E in H1-depleted larvae is corroborated by chromatin IP (ChIP) experiments: the occupancy of both H1 and STAT92E at multiple genomic loci is strongly decreased upon H1 knockdown (Figure 2C).

We also analyzed the distribution of H1 and STAT92E in polytene chromosomes of *hop*^*Tum-l*^ mutant larvae and in animals with STAT92E depleted by RNAi. RNAi-mediated depletion almost completely eliminates STAT92E presence in polytene chromosomes (Additional file 1: Figure S2C). Interestingly, *hop*^*Tum-l*^ mutation also results in reduction and re-distribution of the STAT92E-specific signal in polytene chromosomes (Additional file 1: Figure S2C). In contrast to the effects of H1 depletion on STAT92E distribution, neither STAT92E depletion nor its hyperphosphorylation had a discernable effect on H1 localization (Figure 2D). These results indicate that linker histone H1 strongly contributes to STAT92E tethering to chromatin. H1 is required for the apparent ubiquitous localization of STAT92E and thus may act upstream of STAT92E in regulation of normal chromosome architecture and heterochromatin structure proposed previously [18,19].

### H1 physically interacts with STAT92E

**Figure 3:**
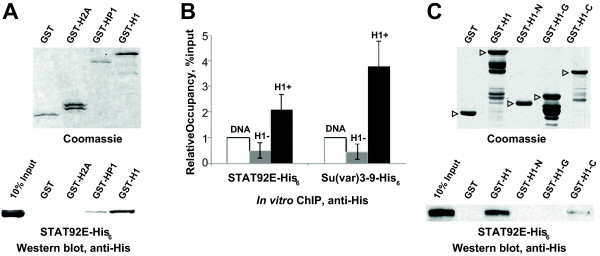
**H1 physically interacts with STAT92E.** Protein-protein interactions between purified STAT92E and H1 were examined *in vitro* by GST pull-down and ChIP. **(A)** GST and GST fusion proteins with full-length H1, HP1 or H2A were expressed and purified from *E. coli* and analyzed by GST pull-down with baculovirus-expressed purified recombinant STAT92E-His_6_. The pull-down samples were examined by SDS-PAGE and Coomassie staining (*top*) or immunoblotting with anti-His_6_ antibody (*bottom*). As a control, 10% of the input STAT92E-His_6_ was examined. **(B)** Binding of STAT92E and Su(var)3-9 to reconstituted chromatin was analyzed by *in vitro* ChIP. Oligonucleosomes were reconstituted on supercoiled plasmid DNA with purified native core histones, with (*H1+*, *dark-gray bars*) or without (*H1–*, *light gray bars*) purified native H1. Non-sequence specific binding to the plasmid (*DNA*, *white bars*) was also examined. His_6_-tagged recombinant proteins were incubated with chromatin/DNA templates, cross-linked, immunoprecipitated with anti-His_6_ antibody and occupancy was measured by real-time PCR of a fragment of the plasmid. The occupancy of proteins relative to input was normalized to occupancy on naked DNA and plotted. The presence of H1 in chromatin templates strongly stimulates binding of both STAT92E and Su(var)3-9*.* All ChIP experiments were performed in duplicate, and each biological sample was analyzed by PCR in triplicate. Error bars represent standard deviation of six experimental points. **(C)** GST and GST fusion proteins with full-length H1, H1 N-terminal domain (H1-N, amino acid residues 1–40), the globular domain (H1-G, residues 41–119) and the C-terminal domain (H1-C, 120–256) were expressed and purified from *E. coli* and used in GST pull-down experiments with baculovirus-expressed purified recombinant STAT92E-His_6_. The pull-down samples were examined as in **(A)**. Full-length polypeptides of GST fusion proteins are indicated by *open triangles*. STAT92E associates with GST fusions of H1 and H1-C but does not interact with GST or GST fusions of H1-N and H1-G.

Since H1 controls localization of STAT92E in chromatin, we next asked if H1 and STAT92E physically interact. Purified recombinant His-tagged STAT92E and a fusion protein of H1 and glutathione-S-transferase (GST) were examined for physical interactions *in vitro* by GST pull-down (Figure 3A). GST-H1 readily interacts with STAT92E as determined by immunoblotting of glutathionine agarose-bound proteins with anti-His antibody. In control experiments, STAT92E did not bind to GST itself or a GST-Histone H2A fusion. Thus, the H1-STAT92E interaction is not due to the high net positive charge of H1 protein or to bridging by potentially contaminating nucleic acids. On the other hand, STAT92E associates with GST-HP1, confirming earlier evidence for an interaction between these two proteins [19]. The observed physical interaction of STAT92E and H1 provides *in vitro* support for a model in which H1 mediates STAT92E recruitment to chromatin.

To further support this model, we examined interactions of H1 and STAT92E in the context of chromatin. To this end, we reconstituted defined oligonuclosomal substrates that did or did not contain H1 [14] and used *in vitro* ChIP to analyze association of purified recombinant STAT92E with H1-containing and H1-free chromatin. The plasmid template used for oligonucleosome reconstitution is not known to contain specific STAT92E recognition sequences. Whereas the presence of nucleosomes inhibited non-specific STAT occupancy at the DNA substrate, addition of H1 to chromatin stimulated STAT92E binding (Figure 3B). Thus, STAT92E physically interacts with H1, both as a free protein and as a component of reconstituted chromatin. These observations are similar to our recent discovery of an interaction between H1 and Su(var)3-9, as well as recruitment of Su(var)3-9 to H1-containing chromatin (Figure 3B) [14].

H1 linker histones consist of a short unstructured N-terminal domain (NTD), a central winged helix-like globular domain (GD) and a long unstructured C-terminal domain (CTD) [1,2]. Particular residues within the GD and regions within the CTD contribute to H1 binding to nucleosomes *in vitro*[26]. To determine which region(s) contribute to the H1-STAT92E interaction, we performed *in vitro* binding experiments with GST fusions of the individual H1 domains and His-tagged STAT92E (Figure 3C). We observed that the H1 CTD interacts with STAT92E, whereas the H1 NTD and GD do not interact with STAT92E. Interaction of full-length H1 with STAT92E appears to be stronger than that of the isolated H1 CTD, suggesting that the structure of the CTD required for interaction with STAT92E may be influenced by one or both of the other H1 domains. Interestingly, we recently found that the CTD of the murine H1d subtype is required for its interactions with DNMT1 and DNMT3B [26]. Thus, the C-terminus of H1 may encompass interaction module(s) for multiple binding partners of linker histone H1.

### H1 suppresses tumorigenesis caused by hyperactive JAK/STAT signaling

**Figure 4:**
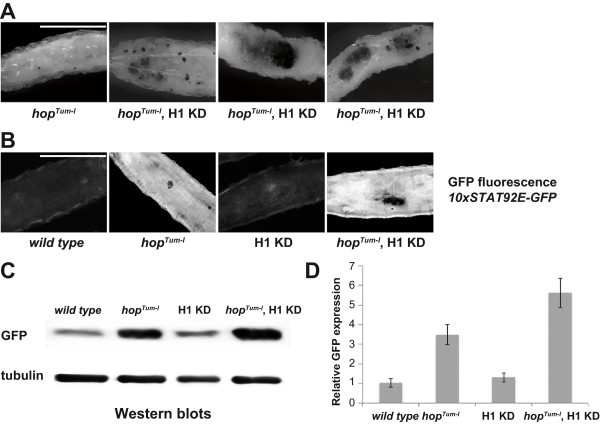
**Depletion of H1 enhances hematopoietic tumor formation caused by hyperactive JAK but does not affect JAK/STAT transcriptional activity. (A)** L3 larvae with a *hop*^*Tum-l*^ mutation (*left*) exhibit hematopoietic tumors (white arrows) when reared at 29°C. When H1 is simultaneously depleted in *hop*^*Tum-l*^ larvae, both the size and the number of tumors are significantly increased throughout the larval body (the three panels on the *right* demonstrate a spectrum of observed phenotypes). For quantitation, see Table 1. *Scale bar* represents 1 mm. **(B)** Homozygous flies that carry an eGFP transgene under control of a promoter with 10 upstream STAT92E binding sites (*10xSTAT92E-GFP*) were mated with appropriate counterparts, *wild type*; *hop*^*Tum-l*^ allele; H1 knockdown (KD) *pINT1-H1*^*4M*^, *Actin-GAL4* allele; or a combined H1 knockdown, *hop*^*Tum-l*^ allele, at 29°C, and eGFP expression was examined in the progeny by GFP autofluorescence. *hop*^*Tum-l*^ mutation but not H1 depletion strongly enhances the expression of eGFP. *Scale bar* represents 1 mm. **(C)** Semi-quantitative western analyses of eGFP reporter expression in whole larvae. *Wild type*; *hop*^*Tum-l*^; *pINT1-H1*^*4M*^, *Actin-GAL4*; and *hop*^*Tum-l*^, *pINT1-H1*^*4M*^, *Actin-GAL4* flies were crossed with *10xSTAT92E92E-GFP* reporter flies, and proteins in lysates of whole L3 larvae in the F1 progeny were analyzed by western blot. Anti-tubulin western was used as a loading control. *H1 KD* H1 knockdown. **(D)** For eGFP quantitation, the GFP staining intensity in western blots (C) was normalized to that of tubulin and plotted. In the *hop*^*Tum-l*^ background, the expression of eGFP reporter is approximately four times higher than that in the *wild type* background and is further stimulated (approximately 1.5-fold) by H1 knockdown, whereas H1 depletion alone does not appreciably affect eGFP expression. The quantitation is based on three independent experiments. *Error bars* represent standard deviation.

In addition to demonstrating that JAK-STAT signaling is involved in heterochromatin stability, Li and colleagues also found that HP1 and Su(var)3-9 can act as suppressors of *hop*^*Tum-l*^-mediated tumorigenesis [18]. The *hop*^*Tum-l*^ allele which causes hyperactive JAK/STAT signaling is associated with a high incidence of neoplastic transformation of larval macrophage-like lamellocytes, resulting in melanotic tumors in a dominant fashion at restrictive temperatures (>25°C) [27]. To investigate a potential role for H1 in *Drosophila* tumorigenesis, we depleted H1 by RNAi in *hop*^*Tum-l*^ mutant larvae. To this end, we crossed flies that carry recombined H1 RNA hairpin and pActin-GAL4 transgenes with *hop*^*Tum-l*^ counterparts. A transgene that depletes an unrelated protein Nautilus [28] was used as a control. When *hop*^*Tum-l*^ larvae underwent H1 depletion at 29°C (to approximately 30% of the wild type level, Additional file 1: Figure S1), we observed a marked increase in the frequency and size of tumors (Figure 4A and Table 1). The tumor index was increased almost twofold by H1 depletion. To confirm a role for H1 in *hop*^*Tum-l*^-mediated tumorigenesis, H1 protein was overexpressed in *hop*^*Tum-l*^ mutant larvae. This was accomplished by combining the *hop*^*Tum-l*^ allele with transgenes encoding full-length H1 and the Actin-GAL4 driver. This combination caused a marked (more than threefold) reduction in the tumor index (Table 1). Taken together with the results of H1 depletion experiments, these data indicate that H1 acts as a suppressor of tumorigenesis caused by hyperactive JAK/STAT signaling.

**Table 1:** H1 regulates hematopoietic tumor formation caused by hyperactive JAK

**Genotype**	***N***	**Tumor index**	***p*** **value**	**H1 expression**
*hop*^*Tum-l*^*/+*	180	0.67		n.d.
*hop*^*Tum-l*^*/+; pINT1-Nau/Act-GAL4*	68	0.69		(100%)
*pINT1-H1*^*6F*^*/+; Tub-GAL4/+*	100	0.00		Approximately 5%
*hop*^*Tum-l*^*/+; pINT1-H1*^*4M*^*/Act-GAL4*	82	1.26	0.005	Approximately 30%
*hop*^*Tum-l*^*/+; UAS-H1/Act-GAL4*	214	0.35	0.003	n.d.
*hop*^*Tum-l*^*/+; UAS-STAT/Act-GAL4*	68	2.76	<0.0001	n.d.
*hop*^*Tum-l*^*/+; UAS-STAT(Y704F)/Act-GAL4*	81	0.67		n.d.

*hop*^*Tum-l*^*/FM7C* flies were mated to wild type flies; *hop*^*Tum-l*^*/FM7C*; *Actin-GAL4/CyO* flies were mated to homozygous transgenic *pINT1-Nau*, *pINT1-H1*^*4M*^, *UAS-H1*, *UAS-STAT92E* or *UAS-STAT92E(Y704F)* flies; homozygous transgenic *pINT1-H1*^*6F*^ flies were mated to *Tubulin-GAL4/TM3, Sb* flies. All crosses were set at 29°C. The tumor index in adult progeny of the appropriate genotype was calculated as described previously [18]. *p* values were calculated by Mann-Whitney test. *N* number of flies scored. Estimated H1 protein expression is presented as percentage (%) of wild type level; *n.d.* not determined.

Importantly, depletion of H1 in the wild type background does not lead to tumorigenesis (Table 1), which suggests that H1 does not play a direct role in the JAK-STAT transcriptional response. To confirm this observation, we made use of a transgenic allele in which a GFP reporter is placed under control of a STAT-responsive promoter containing 10 STAT92E binding sites [29]. We examined GFP expression by GFP autofluorescence of whole larvae or western blot of larval lysates (Figure 4B,C). Upon hyperphosphorylation in the *hop*^*Tum-l*^ mutant, STAT92E becomes transcriptionally active and strongly activates GFP expression. Quantitation of western data indicates about fourfold activation of the transgene upon STAT92E hyperphosphorylation (Figure 4D), which is further increased by H1 depletion in the *hop*^*Tum-l*^ background. On the other hand, H1 depletion alone does not activate transgene expression (Figure 4B,C,D). Thus, H1 does not appear to play a substantial role in direct regulation of normal transcriptional targets of phosphorylated STAT92E.

Rather, our results are consistent with a model in which H1 is required to maintain sequence-independent, ectopic localization of STAT in chromatin. Upon H1 depletion, excess STAT92E is released from the ectopic sites. The released STAT is in an unphosphorylated, transcriptionally inactive form and is unable to activate STAT-responsive genes (Figure 4). On the other hand, in the presence of the mutant *hop*^*Tum-l*^ allele, the STAT released by H1 depletion becomes available as a substrate for phosphorylation by hyperactive JAK, which results in additional activation of endogenous STAT targets and increased tumorigenesis. If this model is correct, then overexpression of STAT itself in the *hop*^*Tum-l*^ mutant should also result in a similar, enhanced tumor formation by virtue of an increased JAK substrate availability. Indeed, as expected, UAS-controlled STAT92E overexpression driven by Actin-GAL4 leads to a fourfold increase of the tumor index in the *hop*^*Tum-l*^ background (Table 1). However, when a non-phosphorylatable mutant of STAT, STAT92E(Y704F) [30], is overexpressed under similar conditions, the tumorigenic effect of *hop*^*Tum-l*^ is not affected.

Ectopic overexpression of either HP1 or Su(var)3-9 under control of a heat shock promoter can also reduce the tumor index in *hop*^*Tum-l*^ mutant larvae [18]. To determine whether H1 is required for the tumor suppressor functions of HP1 and Su(var)3-9, we simultaneously depleted H1 and overexpressed Su(var)3-9 or HP1 in *hop*^*Tum-l*^ larvae. Depletion of H1 abolished the reduction in tumorigenicity caused by overexpression of either Su(var)3-9 or HP1 (about threefold), resulting in tumor indices that are nearly identical to that observed in the original *hop*^*Tum-l*^ allele (Table 2). These observations demonstrate that H1 is required for the tumor suppressor activity of HP1 and Su(var)3-9. They further support a model in which H1 lies upstream of STAT92E, HP1 and Su(var)3-9 in both maintenance of heterochromatin structure and tumorigenesis caused by hyperactive JAK/STAT signaling.

**Table 2:** The tumor suppressor function of HP1 and Su(var)3-9 is dependent on H1

**Genotype**	***N***	**Tumor index**	***p*** **value**
*hop*^*Tum-l*^*/+*	180	0.67	
*hop*^*Tum-l*^*/+*; *ht-HP1/+*	169	0.21	
*hop*^*Tum-l*^*/+*; *ht-HP1/pINT1-H1*^*4M*^, *Act-GAL4*	113	0.62	<0.0001
*hop*^*Tum-l*^*/+*; *ht-Su(var)3-9/+*	224	0.19	
*hop*^*Tum-l*^*/+*; *ht-Su(var)3-9/pINT1-H1*^*4M*^, *Act-GAL4*	93	0.61	<0.0001

*hop*^*Tum-l*^*/FM7C* flies were mated to *wild type* or homozygous transgenic *ht-HP1* or *ht-Su(var)3-9* flies; *hop*^*Tum-l*^*/FM7C*; *pINT1-H1*^*4M*^, *Actin-GAL4/CyO* flies were mated to homozygous transgenic *ht-HP1* or *ht-Su(var)3-9* flies. All crosses were set at 29°C. The tumor index in adult progeny of the appropriate genotype was calculated as described previously [18]. *p* values were calculated by Mann-Whitney test. *N* number of flies scored.

### H1 and STAT92E cooperate in the establishment of heterochromatin structure

H1 depletion results in profound changes of polytene chromosome architecture and heterochromatin structure, activity, and biochemical composition. For instance, H1-depleted larvae largely lose the pericentric H3K9 dimethyl mark (Figure 1) and [13]. However, we previously observed that overexpression of the H3K9-specific HMT, Su(var)3-9, partially ameliorates this defect [14].

The results of this and other studies [18,19] suggest that ectopic localization of unphosphorylated STAT92E in chromatin may play an important role in proper polytene chromosome morphology in larvae, specifically the formation of heterochromatic chromocenter. The ectopic localization of STAT requires linker histone H1, depletion of which brings about STAT92E release from ectopic sites and simultaneous disruption of the chromocenter. Thus, in turn, it is possible that H1-mediated effects on heterochromatin structure may depend, at least in part, on STAT92E.

**Figure 5:**
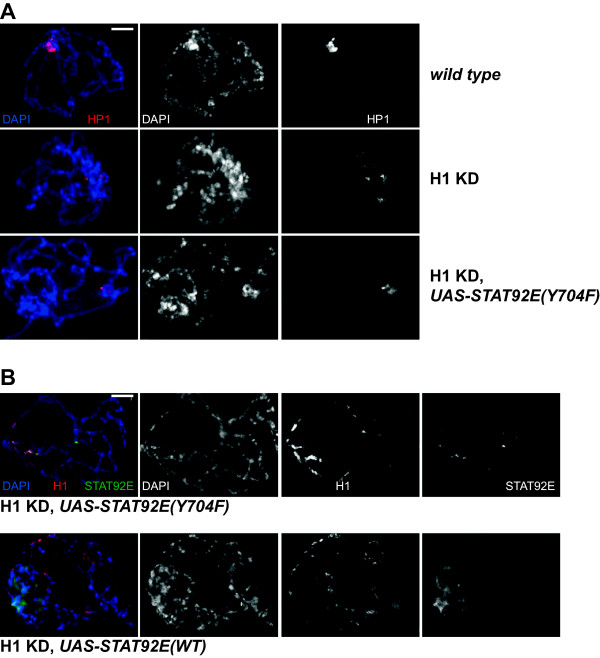
**STAT92E contributes to H1-dependent heterochromatin formation.** Polytene chromosomes of salivary gland cells from L3 larvae were analyzed by indirect immunofluorescence (IF) staining with antibodies against HP1 or H1 (*red*) and STAT92E (*green*). DNA was stained with DAPI (*blue*). *Scale bar* represents 10 μm. **(A)** Polytene chromosome structure in wild type larvae (*top*), H1-depleted larvae (*middle*), and H1-depleted larvae that overexpress nonphosphorylatable STAT, STAT92E(Y704F) (*bottom*). HP1 signal is strongly enriched in a single chromocenter region in the wild type. The chromocenter is not discernable (DAPI), and HP1 staining is dispersed in multiple foci upon H1 depletion (see also Figure 1A). The phenotype is partially rescued by STAT92E(Y704F) expression. **(B)** STAT92E(Y704F) overexpressed in H1-depleted larvae, co-localizes with residual H1 in polytene chromosomes (*top*). Ectopically overexpressed transgenic wild type (*WT*) STAT92E fails to restore the single chromocenter and does not co-localize with residual H1 (*bottom*).

We decided to examine polytene chromosome structure in H1-depleted larvae that overexpress a non-phosphorylatable form of STAT92E(Y704F) *in vivo* in larvae. We discovered that whereas H1 depletion alone results in dissociation of a single chromocenter into multiple foci in close to 100% of examined specimens (Figures 1 and 5A), STAT92E(Y704F) overexpression partially reverses this defect (Figure 5A). In H1-depleted animals that overexpress STAT92E(Y704F), up to 40% of salivary gland cells contain a single chromocenter discernable by anti-HP1 or DAPI staining. Furthermore, when overexpressed, STAT92E(Y704F) re-populates ectopic sites in polytene chromosomes, from which the endogenous STAT92E is evicted due to H1 depletion (compare Additional file 1: Figure S2C and Figure 5B, top), and co-localizes with residual H1. These results indicate that eviction of STAT92E is required for complete penetrance of polytene chromosome defects associated with H1 depletion. They also parallel our previous findings of partial reversal of H1 depletion effects by overexpression *in vivo* of another H1 recruitment partner, Su(var)3-9. In contrast, overexpression of wild type STAT92E in H1-depleted larvae fails to restore the single chromocenter of polytene chromosomes, and transgenic STAT92E does not strongly co-localize with residual H1 (Figure 5B, *bottom*). These observations suggest that overexpressed wild type STA92E is not efficiently tethered to residual H1 in chromatin and does not re-populate ectopic chromosomal loci. Such findings may be explained by the ability of wild type STAT92E to become phosphorylated by JAK kinase and thus capable to bind to native, sequence-specific (and H1-independent) sites. On the other hand, STAT92E(Y704F) is less dynamic, cannot be phosphorylated by JAK kinase or interact with normal targets of active (phosphorylated) STAT and therefore, is more likely to strongly compete with endogenous STAT92E for limiting H1 under conditions of H1 depletion.

**Figure 6:**
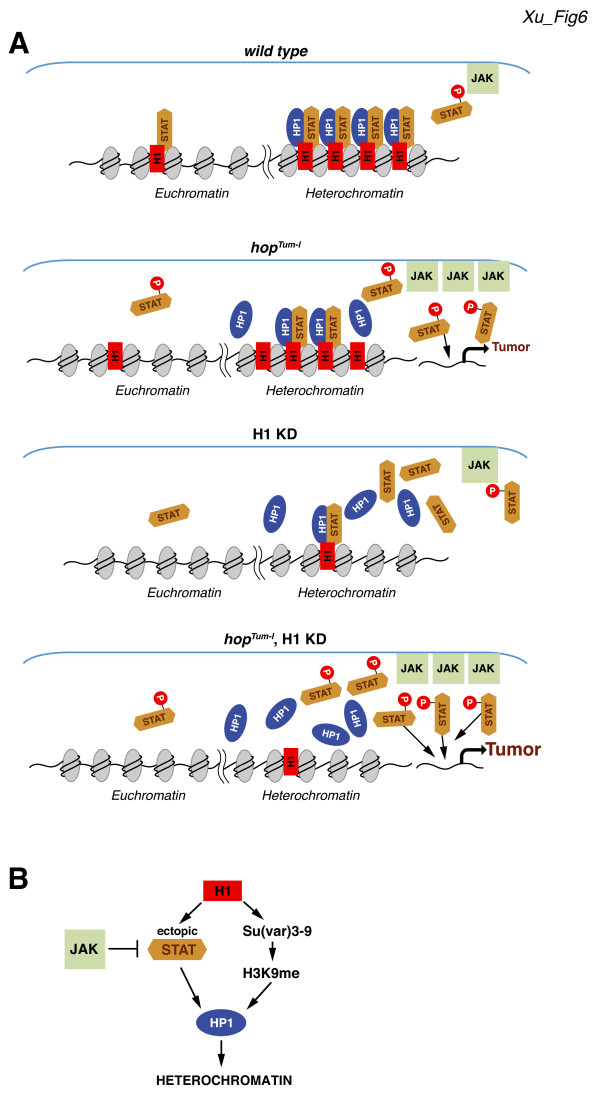
**H1 depletion prevents STAT association with ectopic sites and enhances blood tumor formation induced by hyperactive JAK.** Nucleosomes, H1, HP1, STAT, and JAK are represented by light-gray ovals, red rectangles, blue ovals, orange hexagons, and light-green rectangles, respectively. Hyperactive JAK is represented by an increased number of corresponding rectangles. **(A)***Top*, in wild type chromatin, unphosphorylated STAT92E physically interacts with H1 and is recruited to ectopic loci irrespective of sequence-specific DNA recognition. The two proteins stabilize the association of HP1 with heterochromatin. *Second*, hyperactive JAK in *hop*^*Tum-l*^ larvae phosphorylates a greater fraction of STAT92E, which prevents its association with H1 and HP1 at ectopic sites and destabilizes HP1 association with pericentric heterochromatin. The excess of phosphorylated STAT92E abnormally stimulates downstream transcriptional targets and leads to blood tumor formation. *Third*, the association of STAT92E and HP1 with heterochromatin is dependent on the presence of H1. When H1 is depleted, both STAT92E and HP1 are dissociated from chromatin. Due to limiting activity of wild type JAK, the excess of STAT92E does not activate transcription and does not cause tumorigenesis. *Bottom*, depleting H1 in *hop*^*Tum-l*^ larvae leads to eviction of STAT92E from ectopic sites. The released STAT92E becomes available for phosphorylation by hyperactive JAK and enhances blood tumor formation. **(B)** Two independent pathways for H1-dependent heterochromatin formation. *Arrows* indicate physical interactions/tethering or an enzymatic reaction (H3K9 methylation). Phosphorylation by JAK prevents STAT accumulation at ectopic loci, including pericentric heterochromatin.

The proposed model of H1 and STAT cooperation in the establishment of pericentric heterochromatin structure might at first seem unlikely, because the abundance of a transcription factor, such as STAT92E, would be expected to be many orders of magnitude lower than that of ubiquitous components of chromatin, such as H1 and HP1, and therefore insufficient in amount to mediate global structural properties of chromosomes. To address this perceived contradiction in our model, we examined the abundance of STAT92E and HP1 in larval and embryonic lysates by semi-quantitative western blotting. In these experiments, we compared the intensity of western bands in native samples and samples that contained defined amounts of recombinant proteins (Additional file 1: Figure S3). We discovered that the relative abundance of STAT92E *in vivo* differs by less than tenfold from that of HP1. Furthermore, IF staining of polytene chromosomes for STAT92E (Figure 2A) clearly shows that STAT92E is present throughout polytene chromosomes with a much broader distribution than that expected of a conventional sequence-specific transcription factor. Thus, STAT92E is a much more abundant component of chromatin than previously recognized and it could well contribute to global chromosome structure as proposed in our model (Figure 6).

### A new paradigm for tumor suppression: linker histone H1 as a molecular reservoir for an oncogenic transcription factor

Li and colleagues have proposed that the oncogenic effect of the *hop*^*Tum-l*^ allele and STAT hyperphosphorylation is a direct consequence of the resulting disruption of heterochromatin, which then causes global defects in gene regulation [18]. A recent study in mammalian cells [31] also proposed a role for unphosphorylated STAT5A in stabilization of heterochromatin and tumor suppression *via* repression of multiple oncogenes. Our results do not support this model. The strongest evidence against the disruption of heterochromatin as a principal cause of tumorigenesis is our finding that H1 depletion produces disruptions in heterochromatin that are comparable to or stronger than those caused by the *hop*^*Tum-l*^ mutation, yet H1 depletion alone does not result in tumorigenesis (Table 1). Also importantly, although overexpression of non-phosphorylatable STAT92E(Y704F) largely restores pericentric heterochromatin in H1-depleted salivary glands (Figure 5A), it does not act as tumor suppressor in *hop*^*Tum-l*^ background (Table 1). Therefore, tumor formation and the heterochromatin structural abnormalities observed in the *hop*^*Tum-l*^ mutant are likely independent phenomena.

Instead, our results are consistent with a model in which linker histone H1 serves as a molecular reservoir for STAT92E. We propose that, normally, unphosphorylated STAT92E resides along with H1 in numerous loci throughout chromosomes, including pericentric heterochromatin, where the two proteins stabilize HP1 binding (Figure 6A, top left). The association of STAT92E with these ectopic loci is dependent on H1, but independent of STAT92E canonical DNA recognition elements. Hyperphosphorylation of STAT92E prevents its efficient association with ectopic sites, directs it to specific DNA elements and causes abnormal transcriptional activation and tumorigenesis (Figure 6A, top right). H1 depletion alone leads to release of unphosphorylated STAT92E from the chromatin reservoir and disruption of normal pericentric structures. However, in the absence of hyperactive JAK, it does not result in tumorigenesis, because the normal level of JAK kinase activity is limiting, and generation of higher levels of activated STAT92E is not achieved (Figure 6A, bottom left). Depleting H1 in the presence of hyperactive *hop*^*Tum-l*^ kinase, though, leads to excessive production of phosphorylated STAT92E and enhanced tumorigenesis (Figure 6A, bottom right).

### Linker histone H1 directs two alternative pathways of heterochromatin formation

We reported previously that *Drosophila* H1 interacts with and recruits Su(var)3-9 to promote heterochromatin formation [14]. The results reported here provide evidence for another, alternative pathway of H1-dependent heterochromatin formation, which involves H1 interaction with STAT92E and its recruitment to ectopic sites in chromatin. Eviction of STAT92E from its chromatin reservoir can be achieved by H1 depletion or STAT hyperphosphorylation. However, although hyperphosphorylation of STAT92E disrupts the structure of pericentric heterochromatin, it does not substantially affect H3K9 dimethylation present in HP1-positive foci (Figure 1). Thus, STAT92E appears to be dispensable for Su(var)3-9 localization or activity. Conversely, a null mutation of *Su(var)3-9* does not affect STAT92E localization in polytene chromosomes (Figure 2B). We conclude that STAT92E function in the establishment or maintenance of heterochromatin structure is independent of H3K9 methylation by Su(var)3-9. On the other hand, H1 directs formation of heterochromatin structures *via* both pathways, one that involves H3K9 dimethylation and the other that utilizes STAT-dependent stabilization of HP1 (Figure 6B).

Our analyses reveal that unphosphorylated STAT92E is an abundant and nearly ubiquitous chromatin component (Figure 2A and Additional file 1: Figure S3). Its level of expression approaches 10%–20% of that of heterochromatin protein HP1, or close to 1 molecule per 100 nucleosomes in the genome [32], much higher than expected for a sequence-specific transcription factor. The storage and sequestering of excess inactive STAT in the nucleus is achieved through association with H1-containing chromatin and allows for rapid activation of the JAK-STAT regulatory cascade by external stimuli. At the same time, STAT92E appears to stabilize particular chromatin conformations, such as pericentric heterochromatin in the chromocenter of larval salivary gland chromosomes in *Drosophila*, and physically interacts with H1 and HP1, heterochromatin components. In the future, it will be interesting to analyze molecular interactions in a putative tripartite STAT-H1-HP1 complex in the context of chromatin and to examine how STAT92E modulates the structure of the chromatin fiber *in vitro*.

## Conclusions

By using polytene chromosome analyses in *Drosophila* salivary gland cells, we performed studies of chromatin defects associated with hyperactivation of STAT. Although a connection between heterochromatin integrity and tumorigenesis by JAK-STAT effectors has been proposed recently [18,19], we discovered a new major connection between STAT and linker histone H1 that alters the existing model of STAT-dependent maintenance of heterochromatin and provides mechanistic insight into its regulation. We provide evidence that STAT92E specifically helps to maintain a particular feature of pericentric heterochromatin, namely the chromocenter region of polytene chromosomes in *Drosophila* larvae. Furthermore, we report direct physical interactions of STAT92E with H1 and HP1, key structural components of heterochromatin, and discern molecular mechanisms of STAT-dependent regulation of heterochromatin formation. These observations lead us to propose a coordinate role for STAT, linker histone H1 and HP1 in the maintenance of heterochromatin integrity. Our studies have also revealed that, as a result of its involvement in STAT-dependent organization of chromatin and sequestering STAT in the nucleus, the linker histone H1 acts to suppress tumorigenesis caused by hyperactive JAK-STAT signaling.

## Methods

### Fly strains and genetics

Flies were grown on standard corn meal, sugar, and yeast medium with Tegosept. Stocks were maintained at 18°C. Crosses were performed in an environmental chamber at 29°C. For polytene chromosome staining, all animals were incubated at 29°C throughout their life cycles. Canton-S flies were used as wild type controls. The following fly stocks were obtained from the Bloomington Stock Center and are described in FlyBase: *hop*^*Tum-l*^, *UAST-STAT92E-GFP*, *UAST-STAT92E-RNAi*, *10xSTAT92E-GFP*, *Actin*-*GAL4/CyO*, *HP1-shRNA*, *Su(var)3-9*^*1*^*/TM3,Sb*, and *Su(var)3-9*^*2*^*/TM3,Sb. ht-HP1* and *ht-Su(var)3-9* flies were generous gifts from Dr. G. Reuter (Martin Luther University, Halle-Wittenberg, Germany). *Nau-RNAi* transgenic flies were generously provided by Dr. B. Paterson (NIH). *UAS-STAT(Y704F)* allele was a generous gift of Dr. W. Li (University of California at San Diego). H1 knockdown was achieved by *pINT1-H1[4 M]* transgene expression driven by *Actin-GAL4*[13].

To analyze the tumor index (TI), *hop*^*Tum-l*^ flies were crossed to Canton-S flies, *ht-HP1* or *ht-Su(var)3-9* flies. Alternatively, *hop*^*Tum-l*^ and *Actin-GAL4/CyO* flies were crossed to *pINT-1-H1*^*4M*^ or *pUAST-H1* flies. *hop*^*Tum-l*^, *ht-HP1* and *hop*^*Tum-l*^, and *ht-Su(var)3-9* flies were crossed to *pINT-1-H1*^*4M*^, *Actin-GAL4/CyO* flies. TI was calculated based on observations from F1 adult flies reared at 29°C as described [18]. *p* values were calculated by the Mann-Whitney *U* test using GraphPad Prism software.

### Immunohistochemistry

Indirect immunofluorescence (IF) analyses of polytene chromosomes were carried out as described [13]. DNA was stained by adding 1.5 μg/ml DAPI (Vectashield, CA, USA) to the mounting medium. The following antisera were used at the indicated dilutions: monoclonal mouse anti-*Drosophila* HP1, C1A9 (1:50, Developmental Studies Hybridoma Bank); goat anti-*Drosophila* STAT, dF-20 (1:50, Santa Cruz Biotechnology); affinity-purified rabbit *Drosophila* H1 antiserum (1:5,000) and affinity-purified rabbit anti-H3K9me2 (1:100, Abcam). Appropriate Cy2- and Cy3-conjugated secondary antibodies (Jackson Immuno Research Laboratories, West Grove, PA, USA) were used at 1:200. Specificity of IF staining was verified by appropriate controls, such as staining with secondary antibodies only and staining of polytene chromosomes from H1 and STAT92E knockdown animals (see for example Figure 5B, and Additional file 1: Figure S2C).

For GFP autofluorescence analyses, *wild type*; *hop*^*Tum-l*^; *pINT1-H1*^*4M*^ and *hop*^*Tum-l*^; and *pINT1-H1*^*4M*^ flies were crossed with flies carrying *10xSTAT92E-GFP* transgene. The F1 L3 larvae were placed on a glass slide and immobilized on ice for 10 min.

Fluorescent images were acquired on a Zeiss Axioplan microscope (Carl Zeiss, Oberkochen, Germany) equipped with Zeiss Digital Microscopy Camera AxioCam ICC1and AxioVision Digital Image Processing Software (Carl Zeiss). Stereoscopic images were acquired on a Zeiss SteREO Discovery V8 microscope (Carl Zeiss).

### Recombinant proteins and GST pull-down

Recombinant *Drosophila* His_6_- and FLAG-tagged HP1 protein was purified as described [33]. Full-length *Drosophila* STAT92E cDNA was amplified by PCR from an EST clone (RE13194) (*Drosophila* Genomic Research Center) and cloned into pFastBac1 vector (Invitrogen) in-frame with a C-terminal His_6_-tag. Details of cloning are available on request. STAT92E-His_6_ baculoviruses were prepared using BacToBac System (Invitrogen). The recombinant STAT92E-His_6_ was synthesized in Sf9 cells and purified by TALON His-Tag Purification Resin (Clontech). GST fusions of H1, H2A, and HP1 were expressed in *E. coli* (BL21(DE3)pLys strain) and purified by glutathione-Sepharose chromatography as described [14]. To prepare GST fusions of H1 globular (amino acid residues 41–119), N- (1–40) and C-terminal (120–256) domains, corresponding PCR products were cloned into in pGEX 4 T-1 (GE Life Sciences). The purified proteins were analyzed by SDS-PAGE, and concentrations were determined by Coomassie staining along with BSA protein mass standards (Pierce).

In GST pull-down assays, purified recombinant STAT92E-His_6_ was incubated with GST or GST fusion proteins and purified on glutathione-Sepharose as described [14]. STAT92E-His_6_ binding to GST fusion proteins was detected by anti-His_6_ western of the pull-down samples. Additionally, the pull-down samples were examined for the presence of GST fusion proteins by SDS-PAGE and Coomassie staining.

### Reconstitution of chromatin and ChIP

Reconstitution of H1-containing and H1-free chromatin was carried out as described [14]. For *in vitro* ChIP analyses, approximately 0.5 pmol purified STAT92E-His_6_ or Su(var)3-9-His_6_ protein was incubated with 0.2 pmol supercoiled plasmid DNA (3.2 kb), H1-containing or H1-free chromatin in 20 μl of reaction buffer (50 mM Tris–HCl, pH 7.9, 5 mM MgCl_2_, 4 mM DTT and 2 μg/ml BSA) for 15 min at 27°C. The material was cross-linked for 10 min at room temperature, and the cross-linking was terminated by addition of 9.8 μl of 2.5 M glycine. The material was incubated with 2 μl rabbit polyclonal anti-His_6_ antibody, ChIP grade (Abcam) in 400 μl reaction buffer overnight at 4°C. After immunoprecipitation and cross-link reversal, the DNA was isolated by QIAquick PCR purification kit (Qiagen, Valencia, Santa Clarity, CA, USA). Samples were analyzed quantitatively by real-time PCR (ViiA™ 7 system, Applied Biosystems, Grand Island, NY, USA) as described [13,14]. For H1 and STAT92E qChIP *in vivo*, chromatin was prepared from H1-depleted and control whole larvae, immunoprecipitated as described above and analyzed by real-time PCR as described previously [13,14]. Primer sequences are available upon request. Each sample was analyzed in three independent real-time PCR reactions.

### Immunoblot analyses

Semi-quantitative western analyses of H1, tubulin, and GFP in *Drosophila* salivary gland or whole larval lysates were carried out as described [13]. For quantitation of STAT92E *in vivo*, *Drosophila* embryonic SK (Soeller-Kornberg) extracts were prepared as described [34]. SK extract was boiled in Laemmli loading buffer for 5 min and centrifuged. An aliquot of SK extract was loaded on a 10% SDS-PAGE gel, along with 0.2–20 pmol purified His_6_-tagged STAT92E or 2–200 pmol purified His_6_- and FLAG-tagged *Drosophila* HP1 protein. The following primary antibodies were used at the indicated dilutions: rabbit anti-*Drosophila* H1 (1:5,000); mouse monoclonal anti-tubulin, E7 (1:500; Developmental Studies Hybridoma Bank); mouse anti-GFP (1:1,000, Santa Cruz Biotechnology); mouse anti-*Drosophila* HP1, C1A9 (1:3,000), and goat anti-*Drosophila* STAT dF-20 (1:50). The infrared dye-labeled secondary antibodies were used at 1:10,000 (LI-COR Bioscience, Lincoln, NE, USA). Images were obtained and quantitated using the LI-COR Odyssey Infrared Imaging System.

## Abbreviations

DAPI: 4′,6-diamidino-2-phenylindole; DTT: dithiothreitol; GFP: green fluorescent protein; GST: glutathione S-transferase; HMT: histone methyltransferase; HP1: heterochromatin protein 1; IF: indirect immunofluorescence; JAK: Janus kinase; KD: knockdown; PCR: polymerase chain reaction; qChIP: quantitative chromatin immunoprecipitation; RNAi: RNA interference; STAT: signal transducer and activator of transcription; TI: tumor index; UAS: upstream activation sequence.

## Competing interests

The authors declare that they have no competing interests.

## Authors' contributions

NX carried out and interpreted genetic, immunocytological, and biochemical experiments and drafted the manuscript. AVE performed biochemical experiments. DVF and AIS conceived of the study, participated in its design and coordination, evaluated and interpreted the data, and wrote the final version of the manuscript. All authors read and approved the final manuscript.

## Supplementary Material

Additional file 1 **Supplemental figure legends. Figure S1.** Depletion of H1 protein by RNAi *in vivo* in *Drosophila* larvae. H1 protein levels were examined by semi-quantitative western blotting of salivary gland lysates from *wild type*, *hop*^*Tum-l*^/+, and *pINT-H1*^*4M*^, *Actin-GAL4/CyO* larvae. Whereas *hop*^*Tum-l*^ does not substantially affect H1 levels, ubiquitous GAL4-driven RNAi results in approximately 70% decrease of the expression level. Numbers at the bottom indicate H1 expression relative to *wild type* (100%). **Figure S2.** Changes in distribution of H1, STAT92E, and heterochromatin markers in polytene chromosomes upon depletion of H1, STAT92E, HP1, mutation of Su(var)3-9, or hyperactivation of JAK. Polytene chromosomes of salivary gland cells from L3 larvae were analyzed by indirect immunofluorescence (IF) staining with antibodies against H1, HP1, or H3K9Me2 (*red*) and STAT92E (*green*). DNA was stained with DAPI (*blue*). *Scale bars* represent 10 μm. **(A)** Genome-wide localization of H1 in H1-depleted, *Su(var)3-9* mutant and HP1-depleted polytene chromosomes. H1 depletion (to approximately 30% wild type level, Additional file 1: Figure S1) strongly reduces H1 staining. **B)** Reduced heterochromatin marks in *Su(var)3-9* mutant and HP1-depleted polytene chromosomes. In *Su(var)3-9*[1]*/Su(var)3-9*[2] salivary glands, pericentric heterochromatin-specific H3K9Me_2_ staining is strongly reduced (compare to Figure 1). HP1 staining of polytene chromosomes is completely eliminated upon HP1 depletion by RNAi. HP1 KD, HP1 knockdown. **(C)** Genome-wide localization of STAT92E in polytene chromosomes upon STAT92E depletion and in *hop*^*Tum-l*^ mutants. STAT92E is almost completely eliminated by STAT92E depletion in larvae. *hop*^*Tum-l*^ mutation slightly affects the abundance and localization pattern of STAT92E compared to that in *wild type* chromosomes (Figure 2A). STAT92E KD, STAT92E knockdown. **Figure S3.** Relative abundance of STAT92E and HP1 in *Drosophila* embryonic nuclear extract. *Drosophila* embryo SK extract [33] (see Methods) was analyzed for relative abundance of STAT92E and HP1 by semi-quantitative western blot. The amounts (pmoles) of purified recombinant STAT92E or HP1 proteins were indicated. Click here for file

## References

[B1] Van Holde RE Chromatin 1988 New York: Springer Verlag

[B2] Wolffe AP Chromatin: Structure and function 1998 New York: Academic Press

[B3] Woodcock CL Skoultchi AI Fan Y Role of linker histone in chromatin structure and function: H1 stoichiometry and nucleosome repeat length Chromosome Res 2006 14 17 25 10.1007/s10577-005-1024-3 16506093

[B4] Wolffe AP Histone H1 Int J Biochem Cell Biol 1997 29 1463 1466 10.1016/S1357-2725(97)00026-5 9570139

[B5] Ramakrishnan V Histone H1 and chromatin higher-order structure Crit Rev Eukaryot Gene Expr 1997 7 215 230 10.1615/CritRevEukarGeneExpr.v7.i3.20 9399071

[B6] Misteli T Gunjan A Hock R Bustin M Brown DT Dynamic binding of histone H1 to chromatin in living cells Nature 2000 408 877 881 10.1038/35048610 11130729

[B7] Lever MA Th'ng JP Sun X Hendzel MJ Rapid exchange of histone H1.1 on chromatin in living human cells Nature 2000 408 873 876 10.1038/35048603 11130728

[B8] Felsenfeld G Groudine M Controlling the double helix Nature 2003 421 448 453 10.1038/nature01411 12540921

[B9] Eissenberg JC Reuter G Cellular mechanism for targeting heterochromatin formation in Drosophila Int Rev Cell Mol Biol 2009 273 1 47 19215901 10.1016/S1937-6448(08)01801-7

[B10] Elgin SC Grewal SI Heterochromatin: silence is golden Curr Biol 2003 13 R895 898 10.1016/j.cub.2003.11.006 14654010

[B11] Rea S Eisenhaber F O'Carroll D Strahl BD Sun ZW Schmid M Opravil S Mechtler K Ponting CP Allis CD Jenuwein T Regulation of chromatin structure by site-specific histone H3 methyltransferases Nature 2000 406 593 599 10.1038/35020506 10949293

[B12] Lachner M O'Carroll D Rea S Mechtler K Jenuwein T Methylation of histone H3 lysine 9 creates a binding site for HP1 proteins Nature 2001 410 116 120 10.1038/35065132 11242053

[B13] Lu X Wontakal SN Emelyanov AV Morcillo P Konev AY Fyodorov DV Skoultchi AI Linker histone H1 is essential for Drosophila development, the establishment of pericentric heterochromatin, and a normal polytene chromosome structure Genes Dev 2009 23 452 465 10.1101/gad.1749309 19196654 PMC2648648

[B14] Lu X Wontakal SN Kavi H Kim BJ Guzzardo PM Emelyanov AV Xu N Hannon GJ Zavadil J Fyodorov DV Skoultchi AI Drosophila H1 regulates the genetic activity of heterochromatin by recruitment of Su(var)3-9 Science 2013 340 78 81 10.1126/science.1234654 23559249 PMC3756538

[B15] Daujat S Zeissler U Waldmann T Happel N Schneider R HP1 binds specifically to Lys26-methylated histone H1.4, whereas simultaneous Ser27 phosphorylation blocks HP1 binding J Biol Chem 2005 280 38090 38095 10.1074/jbc.C500229200 16127177

[B16] Nielsen AL Oulad-Abdelghani M Ortiz JA Remboutsika E Chambon P Losson R Heterochromatin formation in mammalian cells: interaction between histones and HP1 proteins Mol Cell 2001 7 729 739 10.1016/S1097-2765(01)00218-0 11336697

[B17] Trojer P Zhang J Yonezawa M Schmidt A Zheng H Jenuwein T Reinberg D Dynamic histone H1 isotype 4 methylation and demethylation by histone lysine methyltransferase G9a/KMT1C and the jumonji domain-containing JMJD2/KDM4 proteins J Biol Chem 2009 284 8395 8405 10.1074/jbc.M807818200 19144645 PMC2659197

[B18] Shi S Calhoun HC Xia F Li J Le L Li WX JAK signaling globally counteracts heterochromatic gene silencing Nat Genet 2006 38 1071 1076 10.1038/ng1860 16892059 PMC3092431

[B19] Shi S Larson K Guo D Lim SJ Dutta P Yan SJ Li WX Drosophila STAT is required for directly maintaining HP1 localization and heterochromatin stability Nat Cell Biol 2008 10 489 496 10.1038/ncb1713 18344984 PMC3083919

[B20] Yan SJ Lim SJ Shi S Dutta P Li WX Unphosphorylated STAT and heterochromatin protect genome stability FASEB J 2011 25 232 241 10.1096/fj.10-169367 20847228 PMC3005427

[B21] Perrimon N Mahowald AP l(1)hopscotch, a larval-pupal zygotic lethal with a specific maternal effect on segmentation in Drosophila Dev Biol 1986 118 28 41 10.1016/0012-1606(86)90070-9 3095163

[B22] Yan R Small S Desplan C Dearolf CR Darnell JE Jr Identification of a Stat gene that functions in Drosophila development Cell 1996 84 421 430 10.1016/S0092-8674(00)81287-8 8608596

[B23] Hou XS Melnick MB Perrimon N Marelle acts downstream of the Drosophila HOP/JAK kinase and encodes a protein similar to the mammalian STATs Cell 1996 84 411 419 10.1016/S0092-8674(00)81286-6 8608595

[B24] Hanratty WP Dearolf CR The Drosophila tumorous-lethal hematopoietic oncogene is a dominant mutation in the hopscotch locus Mol Gen Genet 1993 238 33 37 8479437 10.1007/BF00279527

[B25] Luo H Hanratty WP Dearolf CR An amino acid substitution in the Drosophila hopTum-l Jak kinase causes leukemia-like hematopoietic defects EMBO J 1995 14 1412 1420 7729418 10.1002/j.1460-2075.1995.tb07127.x PMC398227

[B26] Zhou BR Feng H Kato H Dai L Yang Y Zhou Y Bai Y Structural insights into the histone H1-nucleosome complex Proc Natl Acad Sci USA 2013 110 19390 19395 10.1073/pnas.1314905110 24218562 PMC3845106

[B27] Harrison DA Binari R Nahreini TS Gilman M Perrimon N Activation of a Drosophila Janus kinase (JAK) causes hematopoietic neoplasia and developmental defects EMBO J 1995 14 2857 2865 7796812 10.1002/j.1460-2075.1995.tb07285.x PMC398404

[B28] Wei Q Rong Y Paterson BM Stereotypic founder cell patterning and embryonic muscle formation in Drosophila require nautilus (MyoD) gene function Proc Natl Acad Sci USA 2007 104 5461 5466 10.1073/pnas.0608739104 17376873 PMC1838484

[B29] Bach EA Ekas LA Ayala-Camargo A Flaherty MS Lee H Perrimon N Baeg GH GFP reporters detect the activation of the Drosophila JAK/STAT pathway in vivo Gene Expr Patterns 2007 7 323 331 10.1016/j.modgep.2006.08.003 17008134

[B30] Karsten P Plischke I Perrimon N Zeidler MP Mutational analysis reveals separable DNA binding and trans-activation of Drosophila STAT92E Cell Signal 2006 18 819 829 10.1016/j.cellsig.2005.07.006 16129580

[B31] Hu X Dutta P Tsurumi A Li J Wang J Land H Li WX Unphosphorylated STAT5A stabilizes heterochromatin and suppresses tumor growth Proc Natl Acad Sci USA 2013 110 10213 10218 10.1073/pnas.1221243110 23733954 PMC3690839

[B32] Lu BY Emtage PC Duyf BJ Hilliker AJ Eissenberg JC Heterochromatin protein 1 is required for the normal expression of two heterochromatin genes in Drosophila Genetics 2000 155 699 708 10835392 10.1093/genetics/155.2.699 PMC1461102

[B33] Emelyanov AV Konev AY Vershilova E Fyodorov DV Protein complex of Drosophila ATRX/XNP and HP1a is required for the formation of pericentric beta-heterochromatin in vivo J Biol Chem 2010 285 15027 15037 10.1074/jbc.M109.064790 20154359 PMC2865330

[B34] Soeller WC Poole SJ Kornberg T In vitro transcription of the Drosophila engrailed gene Genes Dev 1988 2 68 81 10.1101/gad.2.1.68 3356339

