# Supplementary material for: Drosophila linker histone H1 coordinates STAT-dependent organization of heterochromatin and suppresses tumorigenesis caused by hyperactive JAK-STAT signaling
Source: Epigenetics Chromatin. 2014 Jul 28;7:16. doi: 10.1186/1756-8935-7-16 (PMC4149798; doi:10.1186/1756-8935-7-16)
Supplement: Additional file 1 — Supplemental figure legends. Figure S1. Depletion of H1 protein by RNAi in vivo in Drosophila larvae. H1 protein levels were examined by semi-quantitative western blotting of salivary gland lysates from wild type, hop Tum-l /+, and pINT-H1 4M , Actin-GAL4/CyO larvae. Whereas hop Tum-l does not substantially affect H1 levels, ubiquitous GAL4-driven RNAi results in approximately 70% decrease of the expression level. Numbers at the bottom indicate H1 expression relative to wild type (100%). Figure S2. Changes in distribution of H1, STAT92E, and heterochromatin markers in polytene chromosomes upon depletion of H1, STAT92E, HP1, mutation of Su(var)3-9, or hyperactivation of JAK. Polytene chromosomes of salivary gland cells from L3 larvae were analyzed by indirect immunofluorescence (IF) staining with antibodies against H1, HP1, or H3K9Me2 (red) and STAT92E (green). DNA was stained with DAPI (blue). Scale bars represent 10 μm. (A) Genome-wide localization of H1 in H1-depleted, Su(var)3-9 mutant and HP1-depleted polytene chromosomes. H1 depletion (to approximately 30% wild type level, Additional file 1: Figure S1) strongly reduces H1 staining. B) Reduced heterochromatin marks in Su(var)3-9 mutant and HP1-depleted polytene chromosomes. In Su(var)3-9[1]/Su(var)3-9[2] salivary glands, pericentric heterochromatin-specific H3K9Me2 staining is strongly reduced (compare to Figure 1). HP1 staining of polytene chromosomes is completely eliminated upon HP1 depletion by RNAi. HP1 KD, HP1 knockdown. (C) Genome-wide localization of STAT92E in polytene chromosomes upon STAT92E depletion and in hop Tum-l mutants. STAT92E is almost completely eliminated by STAT92E depletion in larvae. hop Tum-l mutation slightly affects the abundance and localization pattern of STAT92E compared to that in wild type chromosomes (Figure 2A). STAT92E KD, STAT92E knockdown. Figure S3. Relative abundance of STAT92E and HP1 in Drosophila embryonic nuclear extract. Drosophila embryo SK extract [33] (see Methods) [file 1756-8935-7-16-S1.pdf]

SUPPLEMENTAL FIGURE LEGENDS

Supplemental Figure S1. Depletion of H1 protein by RNAi in vivo in *Drosophila* larvae.

H1 protein levels were examined by semi-quantitative western blotting of salivary gland lysates from *wild type*, hop^{Tum-l}/+ and *pINT-H1*^{4M}, *Actin-GAL4/CyO* larvae. Whereas hop^{Tum-l} gain of function mutation does not substantially affect H1 levels, ubiquitous GAL4-driven RNAi results in ~70% decrease of the expression level. Numbers at the bottom indicate H1 expresion relative to *wild type* (100%).

Supplemental Figure S2. Changes in distribution of H1, STAT92E and heterochromatin markers in polytene chromosomes upon depletion of H1, STAT92E, HP1, mutation of Su(var)3-9 or hyperactivation of JAK.

Polytene chromosomes of salivary gland cells from L3 larvae were analyzed by indirect immunofluorescence (IF) staining with antibodies against H1, HP1 or H3K9Me2 (red) and STAT92E (green). DNA was stained with DAPI (blue). Scale bars represent 10 µm.

A. Genome-wide localization of H1 in polytene chromosomes. Polytene chromosomes were prepared from salivary glands of *wild type*, H1-depleted, *Su(var)3-9* mutant and HP1-depleted L3 larvae. H1 depletion (to ~30% wild type level, Suppl. Fig. S1) strongly reduces H1 staining. Effects of *Su(var)3-9* mutation and HP1 depletion on the presence of $H3K9Me_2$ mark and HP1 in polytene chromosomes is examined in Suppl. Fig. S2B. H1 KD, H1 knockdown; HP1 KD, HP1 knockdown.

B. Reduced heterochromatin marks in *Su(var)3-9* mutant and HP1-depleted polytene chromosomes. In *Su(var)3-9[1]/Su(var)3-9[2]* salivary glands, pericentric heterochromatin-specific $H3K9Me_2$ staining is strongly reduced (compare to Fig. 1). HP1 staining of polytene chromosomes is completely eliminated upon HP1 depletion by RNAi. HP1 KD, HP1 knockdown.

C. Genome-wide localization of STAT92E in polytene chromosomes upon STAT92E depletion and in $hop^{Tum\text{-}l}$ mutants. STAT92E is almost completely eliminated by STAT92E depletion in larvae. $hop^{Tum\text{-}l}$ mutation slightly affects the abundance and localization pattern of STAT92E compared to that in *wild-type* chromosomes (Fig. 2A). STAT92E KD, STAT92E knockdown.

Supplemental Figure S3. Relative abundance of STAT92E and HP1 in *Drosophila* embryonic nuclear extract.

Drosophila embryo SK extract [33] (see Methods) was analyzed for relative abundance of STAT92E and HP1 by semi-quantitative western blot. An aliquot of the extract was loaded on an SDS-PAGE gel along with the indicated amounts (pmoles) of purified recombinant STAT92E or HP1 proteins and analyzed by immunoblotting with specific antibodies.

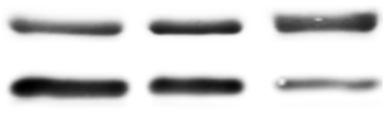

Western blots

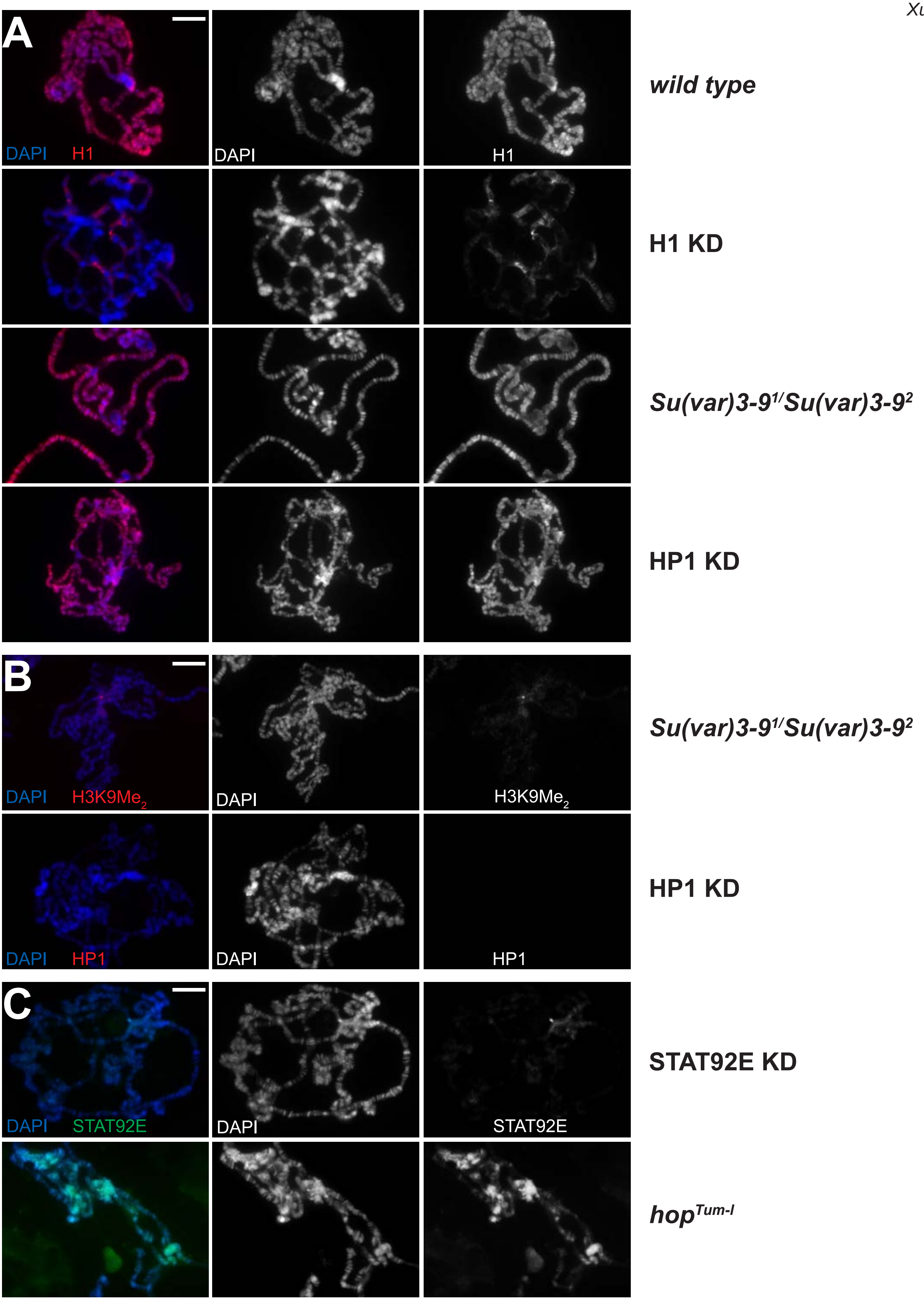
A
DAPI H1
DAPI
H1
wild type
H1 KD
Su(var)3-9^1/Su(var)3-9^2
HP1 KD
B
DAPI H3K9Me2
DAPI
H3K9Me2
Su(var)3-9^1/Su(var)3-9^2
DAPI HP1
DAPI
HP1
HP1 KD
C
DAPI STAT92E
DAPI
STAT92E
STAT92E KD
hop^Tum-l

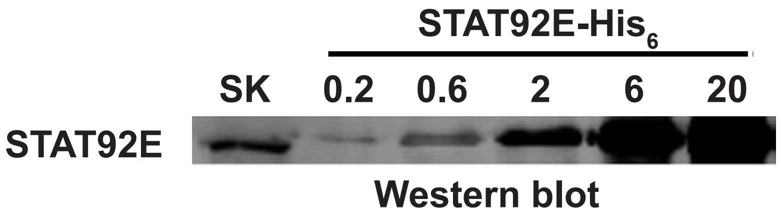
STAT92E-His6
SK 0.2 0.6 2 6 20
STAT92E
Western blot

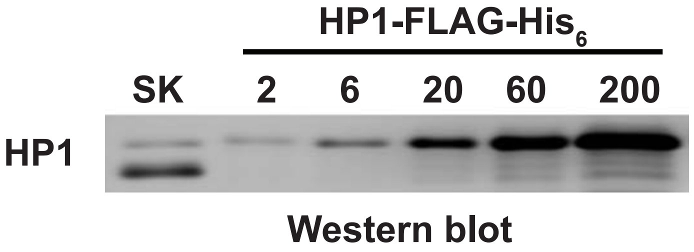
HP1-FLAG-His6
SK 2 6 20 60 200
HP1
Western blot
